# Healthcare provider’s attitude towards disability and experience of women with disabilities in the use of maternal healthcare service in rural Nepal

**DOI:** 10.1186/s12978-017-0330-5

**Published:** 2017-06-29

**Authors:** Hridaya Raj Devkota, Emily Murray, Maria Kett, Nora Groce

**Affiliations:** 1 University College London, 1 – 19 Torrington Place, London, WC1E 6BT UK; 2 University College London (UCL), London, UK; 3 Leonard Cheshire Disability and Inclusive Development Center, Department of Epidemiology and Public Health, University College London (UCL), London, UK; 4 Leonard Cheshire Disability and University College London (UCL), London, UK

**Keywords:** Maternal health, Healthcare providers, Attitude, Disability, Nepal

## Abstract

**Background:**

Women with disabilities are less likely to receive maternal healthcare services compared to women without disabilities. While few studies have reviewed healthcare experience of women with disabilities, no studies have been conducted to understand provider’s attitude towards disability in Nepal, yet the attitude and behaviour of healthcare providers may have a significant influence on aspects of care and the use of service by women with disabilities. This study examines healthcare provider’s attitudes towards disability and explores the experience of women with disabilities in maternal healthcare service utilization during pregnancy and childbirth.

**Method:**

The study used mixed method approach. An attitude survey was conducted among 396 healthcare providers currently working in public health facilities in Rupandehi district of Nepal. For additional insight, eighteen in-depth interviews with women with disabilities who used maternal healthcare services in a healthcare facility within the study district in their last pregnancy were undertaken. The Attitude Towards Disabled Persons (ATDP) scale score was used to measure the attitudes of healthcare providers. For quantitative data, univariate and multivariate analysis using ANOVA was used to understand the association between outcome and independent variables and qualitative analysis generated and described themes.

**Results:**

Mean ATDP score among healthcare providers (78.52; SD = 14.75), was low compared to the normative score of 100 or higher. Nurses/auxiliary nurse midwives obtained the highest mean score (85.59, SD = 13.45), followed by general clinical health workers (Mean score = 82.64, SD 15.10). The lowest score was obtained by Female Community Health Volunteers (FCHV) (Score = 73.75, SD = 13.40) (*P* < 0.001). Younger providers were more positive compared to older age groups (*P* < 0.001). Similarly, providers working in urban health facilities compared to those working in rural health facilities, and non-Dalit providers compared to Dalit providers reported more positive attitudes towards disability (*P* < 0.05). However, there were no significant differences in ATDP mean scores between those who had or had not previously provided services for women with disabilities. The mean score difference between those who received disability training and who did not was also found statistically insignificant (*P* > 0.05). This may reflect the small number of individuals, who have had training on disability thus far, or the nature or quality of the training currently available.

The majority of qualitative interview participants perceived providers to have the negative attitude with poor knowledge, skills and preparation for providing care to persons with disabilities. Few participants perceived the providers as kind, respectful, caring or helpful.

**Conclusion:**

Overall, provider’s attitude towards disability was found to be negative with poor knowledge and skills about providing services. This may have adversely impact maternal healthcare service utilization by women with disabilities. More organized, effective training for healthcare providers is required through on-going mainstream efforts to develop favorable attitudes towards disability. Further research on this subject is also needed.

**Electronic supplementary material:**

The online version of this article (doi:10.1186/s12978-017-0330-5) contains supplementary material, which is available to authorized users.

## Plain English summary

The attitude of healthcare providers may have an influence on aspects of care and the use of service by women with disability. This study examines healthcare provider’s attitudes towards disability and explores the disabled user’s experience in maternal healthcare service utilization during pregnancy and childbirth.

An attitude survey among 396 healthcare providers and eighteen qualitative interviews with women with disabilities who used maternal healthcare services in a healthcare facility within the study district in their last pregnancy was undertaken.

The attitude score among healthcare providers was low compared to the normative score of 100 or higher. Nurse-midwives obtained the highest mean score followed by general health workers. Female community health volunteers obtained the lowest score. Younger providers were more positive compared to older age groups. Providers working in urban health facilities compared to those working in rural health facilities, and non-Dalit providers compared to Dalit providers reported more positive attitude towards disability. No differences were found in scores between those who had or had not previously provided services for women with disabilities and who had received disability training and who did not. The majority of women with disabilities perceived providers to have a negative attitude with poor knowledge and skills for providing care. Few women with disabilities perceived the providers as respectful and caring.

Overall, provider’s attitude towards disability was found negative with poor knowledge and skills providing services. More contact with the person with disabilities and effective training through regular mainstream efforts may help changing provider’s attitude along with increasing knowledge and skills to provide services to women with disabilities.

## Background

People with disabilities, that is people with physical, sensory (i.e. deafness, blindness), intellectual and mental health impairments, often face negative attitudinal barriers within society in general and from healthcare providers in particular while seeking maternal healthcare services [1, 2]. The attitudes and behaviour of healthcare providers obviously have a significant influence on many aspects of care. Negative attitudes of providers may discourage the use of services by the users with disabilities, and negative attitudes may foster low expectations, encourage discriminatory behaviours and marginalization of people with disabilities among health providers themselves [3, 4].

Concerns regarding quality of care are common problems reported by both women with disabilities and without disabilities, largely related to provider’s attitude and behaviours that often discourages women from seeking maternal healthcare services. It is well documented in the literature that women’s perception of quality of care through previous experience during pregnancy to be a key factor in decision making in regards to seeking care subsequently [1, 5].

The literature informs us that people with disabilities often experienced barriers to health services due to provider’s inappropriate attitudes and behaviours [6–8]. Measuring attitudes of health providers towards disability is important to understand their perception so that training for health professionals can be improved in order to foster positive views. A better understanding of the complex relationship between, knowledge, attitude and behaviours would help policy planners to design intervention strategies to change attitudes of healthcare providers towards the person with disabilities and improve healthcare services to those vulnerable groups.

Attitude is defined as the combination of beliefs and feelings held by the individual that predisposes the person to behave in a certain way. It comprises affective, cognitive and behavioural components. Fishbein & Ajzen, (1975) describes attitude in the diagram below, which shows the relationship between people’s attitude, their knowledge and behaviour [9].
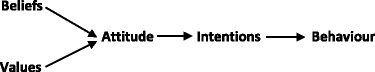


The role of *direct experience* is found particularly important in attitude formation [10–12]. Attitudes are influenced by the individual’s experience combined with positive or negative reinforcement. Attitudes and behaviour are linked; however, attitude is only one factor, social norms and group pressure also influence individual behaviour. Negative attitudes and behaviour come from people not having adequate knowledge as well as negative social norms and group pressure. Demographic factors such as sex, age also influence people’s attitudes [13, 14].

In the literature, a series of studies, primarily from high-income countries have compared the attitudes of health professionals across professions and between professionals and students on disability. A study comparing attitudes among nurses, physiotherapy and occupational therapy students revealed that nurses held the least positive attitude towards disability, while occupational therapy students showed the most positive attitudes [15]. Other studies show personal attributes influencing attitudes towards disability; however, the findings reported are inconsistent. For example, women held more positive attitudes than men in several studies [15–17]. Other studies report mixed findings on the correlation between age of health professionals and attitude [18]. Bakheit and Shanmugalingam (1997) and Dorji and Solomon (2009) found an inverse correlation between age of health professionals and their attitude score [19, 20], while some other studies showed adverse results [21]. In addition, the most influential factor in the formation of attitude was reported to be the intensity of exposure and contact with the person with disabilities [22].

There is a paucity of local research in disability in general and limited attempts has been made to gather information measuring healthcare provider’s attitude towards disability in particular.

A lack of disability-specific knowledge, discomfort working with people with disabilities and misconceptions about disability held by healthcare providers are the key issues contributing to provider’s negative attitude which is a formidable barrier to healthcare services by women with disabilities [23]. These attitudes and misconceptions are often subtle. For example, women with disabilities may not be asked about contraceptives; or healthcare providers might defer a pelvic exam due to the misconception that women with disabilities are generally sexually inactive [6, 24].

Disability issues, care and management are still rarely included in medical school, nor are they usually appropriately addressed in public health and health system management training, particularly in low and middle-income countries. Studies show that while some medical schools in high-income countries are now including disability issues in curricula to improve student’s knowledge, attitude and skills in disability care, it is still not a priority [25].

In recent decades, Nepal has made good progress in maternal health indicators. For example, reduction of MMR from 850 in 1990 to 258 in 2015 [26] with the implementation of the ‘Safe Motherhood Project and Plan 1997 - 2017’. However, improvements in these indicators among marginalized population such as women with disabilities and Dalits are still lower than the general population [27, 28]. As in many low and middle-income countries, Nepal’s healthcare professionals including community health volunteers are the key to providing information and delivery of preventive, clinical and rehabilitative services for all people in the community as well as in health facility settings [29, 30]. However, disability related problems have not attracted policy planners’ attention and there has been no systematic training of healthcare providers in disability care and management. It can be speculated that the continuing poorer results among people with disabilities may reflect inadequate knowledge, misconception and negative attitudes of healthcare providers towards disability.

This study is intended to fill this gap by conducting a mixed-method study that attempts to answer: what are the attitudes of healthcare providers towards disability? Are there any differences in attitudes between professional groups, their exposures to disability and their demographic characteristics (age, gender, etc.)? To provide further insight, we also asked service users about their experience regarding provider’s attitudes towards them.

This study measures healthcare provider’s attitude towards disability in general rather than the specific type of disability, but we recognize that attitudes may vary by disability type. This more detailed question is beyond the scope of this particular study.

## Methods

### Study setting

The study was conducted in Rupandehi district between September 2014 to February 2015. It is located in the southern part of Nepal. Primary healthcare services in the district are delivered through five Primary Healthcare Centers (PHCC), six Urban Health Clinics (UHC), six Health Posts (HP) and 58 Sub-Health Posts (SHP). One district hospital and one zonal hospital (covering six districts) provide secondary care services in the district. In addition to the government health sector, there is a wide network of NGOs and private sector services with private hospitals, nursing homes, clinics and pharmacies/drug shops [31].

### Study design

The study used a simultaneous mixed method approach by which the quantitative and qualitative data were collected at the same time and analyzed in a complementary manner [32]. An attitude survey among maternal healthcare providers along with qualitative interviews with service users was conducted in 2014/15 to examine health service provider’s attitudes towards people with disabilities.

### Sample and sampling procedure

A health facility-linked two-stage approach was used for the selection of healthcare providers. In the first stage, fourty health facilities (50% of the total) were selected from across the district, using criteria that included all types of public health facilities i.e. hospitals and primary level healthcare centers. In the second stage, 396 healthcare providers with a range of jobs from physicians to female community health volunteers (FCHVs) were selected following the criteria recommended by Turner, Angeles, Tsui, Wilkinson, & Magnani, (2001) for health facility (HF) surveys [33]. All healthcare providers were chosen from health facilities having four staff or less, and four from the larger HFs. Similarly, six female community health volunteers linked to each selected urban health facility and five from each rural health facility were chosen randomly. To provide additional insight, a small series of in-depth qualitative interviews were undertaken with eighteen women with disabilities aged fifteen to forty-nine years who had received maternal healthcare service within the last five years. These women were purposively selected and recruited, divided between Dalit and non-Dalit women with disabilities. All healthcare providers and women approached accepted to participate in the study.

### The tool and data collection

Attitude Towards Disabled Person (ATDP) Form B tool, developed in 1960 and updated in 1970 by Yuker, Block, & Campbell was used to measure healthcare provider’s attitude. The tool consists of 30-items with a six rating Likert-type scales. The tool’s reliability coefficient range is estimated as 0.71 to 0.83 [18]. This tool is simple, easy to administer, simple to score and has been used extensively in previous research to assess general attitudes towards disabled persons [16, 34, 35].

The first part of the questionnaire records personal information, the second part consists of the ATDP Form B, and the third part includes three questions about the individual’s contact or exposure to persons with disabilities and any training received about disability. We added the first part (personal information) and the third part (contact or exposure to persons with disabilities) to the original ATDP Form B questionnaire (Additional file 1). Before administrating the survey tools in the field, the instrument was translated into the Nepali language and field-tested to ensure it was comprehensible. An interview schedule (topic guide) was developed and used for the qualitative interviews.

Twelve interviewers were trained for data collection in the field and the researcher (first author) monitored the interviews and data collection process. All forms were checked after completion of interviews; any found incomplete or with entry errors were identified and participants revisited to complete or confirm the information. The first author with the help of two female research assistants conducted the qualitative interviews. All qualitative interviews were audio recorded with participant’s permission.

The construct validity of the survey instrument was checked using ‘Known group technique’ comparing group scores [36] and Cronbach’s Alpha confirmed the internal consistency reliability. For the trustworthiness and confirmability of qualitative data (recording, notes, transcripts), consistency in the process of inquiry, documentation in a reflexive way with a detailed account of the research process and field presentation were followed.

### Measures

Table 1 presents variables, their description and coding used in data analysis. Provider’s ATDP score was the outcome variable for the attitude survey. A list of statement items was read aloud with which the providers expressed agreement or disagreement with each item statement. The participant’s reaction was measured in a response category ranging from +3 to indicate “I agree very much” to −3 to indicate “I disagree very much”. The scale did not have a neutral or zero rating point, forcing participants to make either positive or negative response.

**Table 1:** Variables and their description of measure

Measure	Definition/Coding	Level of measurement
*Outcome Variable*		
Attitude score	Total algebraic sum of the rated scores by the respondents (between 0 and 180)	Ordinal
*Background Variables*		
Respondent’s location	At the time of survey, respondent living in VDC are considered rural and living in Municipality are urban. (1 = rural, 2 = urban)	Categorical
Age	Completed age of women at the time of survey in years. (1 = 18–24, 2 = 25–34, 3 = 35–44, 4 = 45–54, 5 = 55–60)	Categorical
Gender	Male or female respondent (1 = male, 2 = female)	Categorical
Caste/Ethnicity	Self-reported caste and ethnicity of respondent woman (1 = Dalit, 2 = Non-Dalit)	Categorical
Provider’s type	Classification of healthcare providers by their job role. (1 = FCHV, 2 = ANM/Nurse, 3 = Dr./HA/AHW)	Categorical
Provider’s exposure to disability	Service/treatment given to persons with disabilities. (1 = Yes, 2 = No) Maternal health care service given to women with disabilities. (1 = Yes, 2 = No) Disability related training/orientation received. (1 = Yes, 2 = No)	Categorical

The ATDP score ranged from 0 to 180. The score interpretation is based on the individual’s perceived similarity or differences between persons with and without disabilities. A higher score indicates perceiving a person with disabilities as similar to a person without disabilities. A lower score indicates the respondent perceives persons with disabilities as different from persons without disabilities. Higher scores can also be interpreted as an individual displaying a more accepting (positive) attitude towards persons with disabilities, while lower scores reflect a rejecting or discriminatory attitude towards persons with disabilities [37]. There is not consensus in the literature about what threshold is regarded as a positive score. However, scores of 110 for male and 113 for female are set as thresholds [18].

The instrument consisted of both positively and negatively worded items. Following the established methodology for analysis of the ATDP tool [18], the first step of analysis was to change the signs of the items. This was done by changing the signs of the positive items; the algebraic sum of all the item scores was obtained. The sign of the sum then reversed from +ve to –ve and vice versa. To eliminate -ve value, a constant 90 was added to convert all scores to positive. Thus, the resulting scores ranged from 0 to 180, indicating higher scores reflecting a positive attitude.

### Data analysis

Findings presented are from 396 survey interviews of healthcare providers and eighteen qualitative interviews of women with disabilities. The survey data was checked for accuracy and completeness, then entered into the computer in Epi-INFO version 3.4.1 to minimize entry error and imported into SPSS (version 16.0 for Windows) for analysis. Data was then cleaned running frequencies and tabulation and crosschecked for consistency, tallying with the related items. Analysis of variance (ANOVA) statistics was used to compare mean attitude scale score and interaction of background, exposure variables and professional status. Mean comparison and statistical significant was assessed using ANOVA statistics to understand the association between variables. The association was considered significant with *P* < 0.05.

The audio-recorded qualitative interviews were transcribed verbatim in Nepalese and then translated into English. Data were coded and analyzed adopting a grounded theory approach (theme content analysis) that identified concepts and categories. Themes were then grouped into categories and analyzed. Quotes were selected and presented to represent the themes mentioned. Quantitative and qualitative findings were then merged and analyzed to produce the findings and conclusions reported below.

## Results

### Characteristics of survey participants

Table 2 shows selected characteristics of healthcare providers who responded to the ATDP survey. A total 396 providers participated in this survey, and the findings have been collated into three groups based on their role and likely contact with pregnant women with disability. Of these, reflecting the general distribution of healthcare providers in the area, more than half (54.3%) were female community health volunteers (*n* = 215) who are the first contact in providing maternal healthcare service in Nepal’s health delivery system. Approximately 24% (*n* = 94) were auxiliary nurse midwives (ANMs) and nurses who provide the majority of professional maternal care in health services. The remaining 22% (*n* = 87) were other health workers (AHW, HA, doctors) who provide more general medical care, including maternal care. The male: female ratio of the participants was 1:4. The majority (77.3%) of the providers were from rural health facilities (*n* = 306), which consist of health posts, sub-health posts and birthing centers; whereas 22.7% (*n* = 90) participants were from urban health clinics and hospitals. By age, the largest number of providers (85.6%) were between 25 and 54 years (*n* = 339), with a small portion (6.1%) below 25 (*n* = 24), and 8.3% (*n* = 33) above 54. The age of respondents ranged between 18 and 60 years and the mean age was 40. Also reflecting the general distribution of healthcare providers by caste in the region, less than one out of ten providers were Dalits, who are considered as the lowest in the caste hierarchy. This is summarized in the table below:

**Table 2:** Distribution of respondents by selected background characteristics

Background Characteristics	Numbers (Total *n* = 396)	Percent
Respondent’s Location		
Rural	306	77.3
Urban	90	22.7
Respondent’s Age		
18–24 Years	24	6.1
25–34 Years	89	22.5
35–44 Years	135	34.1
45–54 Years	115	29.0
55–60 Years	33	8.3
Respondent’s Gender		
Male	73	18.4
Female	323	81.6
Caste Group		
Dalit	35	8.8
Non Dalit	361	91.2
Provider’s Type		
Dr/HA/AHW	87	22.0
Nurse/ANM	94	23.8
FCHVs	215	54.3
No of Respondent by Health Facility Type		
Hospital	45	11.4
PHCC	48	12.1
HP	27	6.8
SHP	231	58.3
UHC	45	11.4

*FCHV* Female Community Health Volunteer, *ANM* Auxiliary Nurse Midwife, *HA* Health Assistant, *AHW* Auxiliary Health Worker, *PHCC* Primary Health Care Centre, *HP* Health Post, *SHP* Sub-Health Post, *UHC* Urban Health Clinic

### Contact or exposure of healthcare providers to people with disabilities

**Fig. 1:**
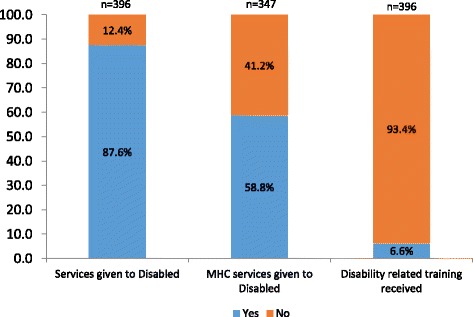
Healthcare provider’s exposure to disability

Survey participants were asked about their exposure/contact to people with disabilities and any training related to disability received before or during their service period. The majority of healthcare providers (87.6%) were found to have been exposed to people with disabilities through the provision of services, and 58.8% have given maternal healthcare services to women with disabilities. Interestingly, only 6.6% of healthcare providers have received some sort of disability-related training (Fig. 1).

### Analysis of survey results

The overall ATDP mean score obtained by the respondents was 78.52 (SD = 14.75) ranging from a minimum score of 38 to the highest of 127. The nurses/ANMs mean score was 85.59 (SD = 13.45), followed by the general clinical health workers (mean score = 82.64, SD 15.10), who ranged from 56 to 127 and 44 to 115 respectively. The lowest score was obtained by FCHVs (mean = 73.75, SD = 13.40) ranging from 38 to 108 (*P* < 0.001). (Table 3).

**Table 3:** ATDP scores by profession type

Provider’s Type	Number	Mean	SD	Range
All	396	78.52	14.75	38–127
Dr/HA/AHW	87	82.64	15.10	44–115
Nurse/ANM	94	85.59	13.45	56–127
FCHVs	215	73.75	13.40	38–108

*Dr* Doctor, *HA* Health Assistant, *AHW* Auxiliary Health Worker, *ANM* Auxiliary Nurse Midwife, *FCHVs* Female Community Health Volunteers

### Analysis of association between some selected factors and ATDP scores

Mean ATDP scores differed by age, gender, caste and provider type. For example, mean ATDP scores were lower for older versus young health providers (*P* < 0.001). Male providers scored higher ATDP scores compared to females (mean = 82.97 vs 77.51, *P* < 0.005). In the caste groups, Dalit providers scored lower than non-Dalits (69.69 vs 79.37, *P* < 0.001). Nurse/auxiliary nurse midwives scored higher compared to doctor/health assistant/auxiliary health workers (85.59 vs 82.64; *P* < 0.001). Female community health volunteers scored the lowest among the providers (mean = 73.75; *P* < 0.001). However, no significant differences found in mean ATDP scores between providers working in rural and urban health facilities (77.8 vs 81.02, *P* > 0.05) (Table 4).

**Table 4:** Analysis of ATDP score by demographic variables

Factors related to attitude	Number	Mean score	SD	*P* – Value
Location	396	78.52	14.97	
Rural	306	77.78	14.97	*P =* 0.066
Urban	90	81.02	13.74	
Age	396	78.52	14.75	
18–24 Years	24	82.96	13.28	*P* = 0.000
25–34 Years	89	83.74	14.74	
35–44 Years	135	76.76	13.84	
45–54 Years	115	77.72	14.78	
55–60 Years	33	71.15	14.77	
Gender	396	78.52	14.75	
Male	73	82.97	15.26	*P* = 0.004
Female	323	77.51	14.46	
Caste	396	78.52	14.75	
Dalit	35	69.69	12.55	*P* = 0.000
Non Dalit	361	79.37	14.68	
Provider’s Type	396	78.52	14.75	
Dr/HA/AHW	87	82.64	15.10	*P* = 0.000
Nurse/ANM	94	85.59	13.45	
FCHVs	215	73.75	13.40	

Table 5 shows slightly higher mean ATDP scores to those who gave maternal healthcare services to women with disabilities than those who did not (79.49 vs 76.35), however, this difference was not significant *P* > 0.05). In addition, there was no difference in the scores between providers who received some sort of disability-related training and those who did not receive any training (79.04 vs 78.48, *P* > 0.05). By contrast, the mean score was lower (mean = 78.20; SD = 14.83) for those who had been exposed to persons with disabilities through the provision of general service or treatment, versus those who were not exposed (78.20 vs 80.78, *P* > 0.05). However, none of the exposure factors was significantly associated with ATDP score.

**Table 5:** Analysis of ATDP score by exposure variables

Exposure variables	Number	Mean	SD	*P* - Value
Service/Treatment given to disabled	396	78.52	14.83	
Yes	347	78.20	14.83	*P* = 0.252
No	49	80.78	14.12	
MHC service given to disabled women	347	78.20	14.83	
Yes	204	79.49	14.75	*P* = 0.052
No	143	76.35	14.79	
Disability related training received	396	78.52	14.75	
Yes	26	79.04	12.86	*P* = 0.852
No	370	78.48	14.87	

## Disabled user’s experience and perception of healthcare provider’s attitude towards them

### Sensitivity and care

Women with disabilities reported mixed experiences and opinions about the provider’s sensitivity and care themselves. Some respondents in their in-depth interview reported a positive experience with the healthcare providers, while others did not. Many reported that the providers were kind, caring and treated them respectfully in a welcoming environment. Several stated that they received counselling and advice in their ANC visits and support during childbirth by the nurses and doctors. One of the women in her interview reflected on her positive experience with providers by saying:

*“They treated nicely and made me understand properly. They would help me lie down themselves….Sister helped me in the health post, and later in the hospital, both doctor and sister helped me.”*

*- A 35 year old Dalit woman with physical disability (Participant # 1) (17* ^*th*^ *February 2015; Kerbani)*

Two other participants reported a similar experience. They stated that providers were helpful and caring towards them during their ANC visits and gave them confidence in delivery:

*“I found health workers happy to see me whenever I went to the hospital. They used to help me, holding my hand while I entered the hospital…they always told me “didi (sister), walk carefully” when the floor of the hospital was damp and slippery.”*

*- A 30 year old woman with physical disability (Participant # 3) (20* ^*th*^ *February 2015; Siktahan)*

*“….. They encouraged me very well. They helped me very much. They consoled me very much and they were very helpful to me every time even I was screaming.”*

*- A 26 year old non-Dalit woman with physical disability (Participant # 10) (11* ^*th*^ *February 2015; Devdaha)*

However, as noted above, not all the participants reported that the providers were sensitive and caring. Some said that providers were discouraging, rude and abusive. Three women with disabilities reported that they were not given complete ANC check-ups or counselling when using services. They added that the nurses frequently scolded and shouted at them during the delivery. Providers in private hospitals were reported to be comparatively polite, and more sensitive and caring than providers at higher/secondary level public hospitals. While being asked about her perception of provider’s skills and behaviour, a disabled participant stated:

*“Well sir, I did not think of who is capable or skilled. All I wanted was to be treated well and polite. I got the impression that the staffs there (at the government hospital) did not give proper care. It was 4-5 years back, I do not know if it is the same now as well.”*

*- A 33 year old non-Dalit woman with visual disability (Participant # 11) (11* ^*th*^ *February 2015; Karahiya)*

The vast majority of the in-depth interview participants reported that providers did not explain things to them properly or give information about their pregnancy while attending antenatal clinics. It was evident that only a very few were advised to go for delivery in a health facility and none of them were informed about the need for post-natal check-ups. One of the participants reported her experience while attending ANC clinic:

*“She didn’t check other things and never explained; only palpated my abdomen and sent me back with some medicines.”*

*- A 27 year old non-Dalit woman with physical disability (Participant # 8) (19* ^*th*^ *February 2015; Saljhundi)*

Another participant reported a similar experience during her first pregnancy check-up in a primary healthcare center:

*“When I went to Dhakdahi PHC they told me to do a urine test to confirm my pregnancy. They did not explain things clearly to me the first time and I returned back to home. The next time I went, the doctor asked me who had checked me before. Once I told him, the doctor was annoyed with his staff, asking why they didn’t check me properly.”*

*- A 30 year old Dalit woman with physical disability (Participant # 3) (20* ^*th*^ *February 2015; Siktahan)*

In addition to the problems faced by other women with disability, all of the blind service users stated that they were not guided or given proper information about where the consultation room was, and overall they found the system in the health facility frustrating:

*“We never got the information. They only made me go from one room to the next. What would be their loss to provide help to their patients?”*

*- A 33 year old non-Dalit woman with visual disability (Participant # 11) (11* ^*th*^ *February 2015; Karahiya)*

Poor care, rude and impolite staff - particularly in public health facilities - were a common experience reported particularly by Dalit and non-Dalit women with disability using the services:

*“I found staff in government hospital rude and not at all polite.....it is for all because I have heard many people say about sisters in the government hospital that they are very rude and treat people bad. Some of them were polite too but all of them were not like that. I might have that impression. Mostly, listening to others as well it is found that sisters in government are more rude and aggressive than in private hospitals nor do they care.”*

*- A 31 year old non-Dalit woman with visual disability (Participant # 12) (11* ^*th*^ *February 2015; Deepnagar)*

### Maintaining distance and avoiding communication

Health practitioners often struggled to understand disabled peoples’ needs as they are not formally trained to provide services to this group. Their attitudes were reported to be distant and uncommunicative by many of the women with disabilities . This discouraged them from seeking services. A number of women with disability reported their disappointment due to the disengagement of the provider. Additional findings from service users revealed that healthcare providers avoided talking to people with disabilities; it may be because they are not sure how to communicate with them. It was common that the providers did not ask the woman directly about her problem, but rather regularly asked whoever had accompanied her; leaving the women with disability feeling ignored.

Some of the interview participants complained about provider’s poor interpersonal and communication skills that often distanced them from the service users with disabilities. For example, a woman with visual disability expressed her frustration when seeking the services – (and it is also notable that she herself sees as being grouped with women with other types of disabilities, and is uncomfortable with being thus addressed):

*“They (providers) used to say I was blind, and behaved as if I was deaf and could not hear them. So they asked my mum …When nobody spoke to me, I thought it was because my mum was there so they did not ask me anything but only to my mum. But after I returned home I started to feel bad; I felt they treated me like someone mentally retarded or deaf, so they asked my mum rather than asking me”.*

*- A 33 year old non-Dalit woman with visual disability (Participant # 11) (11* ^*th*^ *February 2015; Karahiya)*

### Preparedness of health workers for providing care to women with disabilities

The study found providers lacking in knowledge about caring for people with disabilities and also having a poor understanding needs and rights of people with disabilities. Service providers were found to be untrained in specific skills such as communication-related to disability, which would enable them to give better and more targeted services for people with disabilities. Many service users with a disability also perceived that providers had no confidence in treating them. One of the service users recalled her experience concerning the clinical determination of whether she was pregnant or not:

*“I couldn’t get an idea about that at this institution here. One of the sisters was confused whether I was pregnant or not.”*

*- A 30 year old Dalit woman with physical disability (Participant # 3) (20* ^*th*^ *February 2015; Siktahan)*

Another user was not confident with the provider’s skills and ability to handle her delivery:

*“I was very afraid during the delivery time wondering if they could or couldn’t do because I was disabled; if they would understand me or not, and if they could handle me properly or not. I was fearful of all these things.….until the final result I was afraid.”*

*- A 26 year old non-Dalit woman with physical disability (Participant # 10)* *(11* ^*th*^ *February 2015; Devdaha)*

Many women with disabilities reported that the attitudes of providers and their understanding about disability were negative and often discouraging, expressing concern about sexual and reproductive health choices of women with disabilities. Some participants reported that they faced challenges due to preconceived mind-sets and limited understanding about disability and disabled people’s desire and expectations. For example, one of the participants with low vision reported that she was surprised by the doctor’s advice not to have any more children due to her disability. She wondered what different risks she would have than women without disabilities:

*“Doctor suggested me not to have more than one child when I had gone for a check-up of my baby. Due to this, I aborted two pregnancies. They said this baby is healthy and not to take the risk with other pregnancies.”*

*- A 31 year old non-Dalit woman with visual disability (Participant # 12) (11* ^*th*^ *February 2015; Deepnagar)*

The participant’s interview clearly suggests that the health professionals and facilities are poorly prepared and informed to give services to women with disability. Some respondents stated that they faced problems in the government hospital because the providers were not confident about handling their delivery. One of the participants noted:

*“I was there for two days. I was about to deliver and asked them whether it is possible here or not; finally, they said we can’t deliver you here and then I had to go to AMDA (hospital).”*

*- A 35 year old Dalit woman with physical disability (Participant # 1) (17* ^*th*^ *February 2015; Kerbani)*

Many of the participants with disabilities said they avoided public hospitals and preferred to go to private institutions, even though it was costly. They reported that it was not only a matter of provider’s insufficient knowledge and skills to provide the services but also the rude and abusive behaviour towards them in public health facilities that encouraged them to seek services from private providers. A woman with visual disability gave her view for the reason to attend a private hospital:

*“This is the reason why I prefer private hospital rather to government - at least you get service and respect in private for the fee you pay.”*

*- A 33 year old non-Dalit woman with visual disability (Participant # 11) (11* ^*th*^ *February 2015; Kerbani)*

Another participant with visual disability spoke about her bad experience in all health facilities, with no system in the registration to identify women with disabilities, and service providers lacking basic knowledge and skills:

*“……..and the other thing is, the nurses do not know how to hold us while walking. For people with blindness, they should not push (pat) from behind and ask us to walk as we might bang in front somewhere. So, I feel mentioning us as blind in our card would be much helpful.”*

*- A 28 year old non-Dalit woman with visual disability (Participant # 13) (11* ^*th*^ *February 2015: Devdaha)*

## Discussion

The survey results highlighted negative attitudes towards people with disabilities among healthcare providers in the study district. The mean ATDP score for the respondents in this study was found to be significantly lower (mean score 78.5) than the normative score of 113 presented by Yuker Block and Young [18]. The literature reported the provider’s ATDP scores consistently greater than 100 [35].

Lack of knowledge combined with prejudice against people with disabilities may have resulted in stereotyping and negative attitudes among the providers. Previous studies both in Nepal and beyond highlight the fact that provider’s attitudes and behaviours often reflect broader societal prejudices [10, 38]. Providers, particularly those with advanced medical skills, like physicians, often come from upper class, educated communities where social hierarchy further influences assumptions about the social structure, culture and beliefs towards poorer, minority ethnic groups and lower caste people as well as those with a disability [39]. Despite this, this research found inconsistent results in the relationship between the provider’s attitude and some socio-demographic variables. For example, there were slightly higher ATDP scores among healthcare providers working in urban settings; but there was no relationship observed between the place of work and provider’s attitude. Compared to female providers, males had higher ATDP mean scores, indicating that males have more positive attitudes towards people with disabilities. Conversely, nurses and auxiliary nurse midwives (all female) had higher scores than the other two categories of professional groups. Doctors, health assistants and community medicine auxiliaries, both male and female, scored low. Female community health volunteers also scored low.

Of the total female respondents, more than 70% were community health volunteers whose exposure, education, and awareness about disability and disability rights may have been comparatively lower than that of nurses and midwives. Given the higher percentage of respondents with low levels of education and knowledge, the lower ATDP mean score for females might have been over-weighted reflecting the attitudes of female community health volunteers. This is consistent with the literature, which reports inconsistency in regards to gender difference in ATDP scores. However, the vast majority of this research has been conducted in western countries. Moreover, many of the studies in the published literature were conducted in medical and nursing schools. In this body of research, women held more positive attitudes towards people with disabilities [15–17]. In fact, Yuker, Block, & Young, (1970) recommended the normative ATDP score to be higher for female than for male (113 vs 110).

Age and number of years practicing medicine correlated negatively with attitude scores of healthcare workers. The study found that younger healthcare providers were more positive in their attitudes towards people with disabilities than the older providers. This finding was consistent with the findings of a study conducted in South India and Bhutan, but contradicts the findings of studies conducted in Europe and North America [15, 19, 20]. The more positive attitude among younger healthcare providers perhaps indicates a generational change in how disability is viewed, with disability increasingly becoming more culturally acceptable in Asian cultures.

The study also confirmed that provider’s caste significantly correlated with ATDP score. Non-Dalit respondents had more favorable attitudes towards people with disabilities compared to their Dalit counterparts. This indicates that social and cultural beliefs among Dalits that hold a more negative view of disability than among non-Dalits. This may affect negatively on maternal health choices for seeking care for women with disabilities in Dalit households.

Demographic variables such as age, gender, education and place of living have, however, often been reported as insignificant in non-disabled peoples’ attitudes towards people with disabilities [34, 35, 37]. Antonak (1981) reported the most influential factors in attitudinal scores were exposure and the intensity of the contact with people with disabilities [22]. In contrast, this study did not show any correlation between ATDP score and exposure and knowledge variables.

Another interesting finding was that the study did not find attitude differences between the providers who had received disability- related training and those who had not. This finding contradicted the study findings of Cervasio & Fatata-Hall, (2013) conducted among nurses in the United States which examined their attitude before and after disability education [40]; and suggests that short training and exposures may not be enough to change the attitudes of Nepalese healthcare providers towards people with disabilities.

It is also worth noting that the negative attitudes among healthcare providers found in this study may simply reflect general negative attitudes of Nepalese society towards people with disabilities, particularly as the majority of healthcare providers have received no training or awareness interventions to alter broader social attitudes and perceptions.

### User’s experience and perception of healthcare provider’s attitude towards them

The study revealed mixed findings in regards to user’s perception of providers’ attitudes towards women with disabilities. Some of the participants with disabilities in their in-depth interview expressed overall positive experiences with healthcare providers, while others did not. The range of positive attitudes and behaviours displayed towards women with disabilities have been identified in many studies, ranging from being open, friendly, and welcoming to respectful and caring. The most common negative attitudes informed by the literature in relation to healthcare workers were that healthcare workers were disrespectful, abusive, rude, discriminatory, and neglectful [1]. A systematic review found a range of interrelated reasons for these attitudes, with socio-cultural, organizational and individual factors contributing to the attitudes and behaviours of healthcare workers [39].

The literature reports that disrespect, abuse and rudeness is widespread to both women with and without disabilities during facility-based childbirth [41]. While many women with disabilities interviewed were aware that all women both with and without disabilities might be treated poorly, many felt that their disability compounded or intensified this abuse. Moreover, facing disrespect, abuse and rudeness in many areas of their lives these women with disabilities may more hesitant than their peers without disabilities to tackle yet another series of barriers when pregnant and this may be an additional factor in when and where they decide to access healthcare services.

Interestingly, this study also found that negative attitudes and abusive behaviour predominantly among public healthcare providers in higher-level health facilities, rather than among the staff in private health facilities or community-based birthing centers. Amongst the possible explanations for this could be that there is less community involvement in the higher-level healthcare facilities in management and service delivery.

Provider’s negative attitude and abusive behaviors predominating in public health facilities rather than private health facilities were consistent with the findings of Mannava et al. [1]. A recent qualitative study [38] conducted in Nepal among women with disabilities had similar findings in relation to user’s perception towards healthcare provider’s attitudes. However, their study revealed that provider’s negative attitude and abusive behaviours differed according to types of disability, and were experienced more by women with hearing and speech disabilities.

The literature also suggests that similarities exist among healthcare provider’s negative attitude and behaviours in both low and high-income countries. Consistent findings, for example insensitive, abusive health providers with the lack of knowledge, skills and limited information about the needs of people with disabilities were also reported in studies conducted in the US and UK [6, 42, 43].

A broader issue may be that in resource-poor countries, the lack of respectful care from healthcare providers may have due to their dissatisfaction with the healthcare system. The literature suggests that negative attitudes and behaviours of healthcare workers are frequently related to their poor working conditions, which include heavy workloads, long working hours, low pay, shortage of equipment and medicines [1]. Moreover, maternal healthcare providers are often predominantly female, with relatively low status in health system hierarchy and poor salaries. Many of them may have been inadequately trained and supervised at work, and have limited autonomy - yet have great responsibilities. Maternal healthcare providers in Nepal are not excluded from this situation and it maybe that their negative attitude and behaviours in part reflect their dissatisfaction with the Nepalese healthcare system.

### Limitations of study

We acknowledged several limitations associated with this study. The study was a part of a Safe Motherhood Project in Nepal; therefore, the study population was limited to one project district. However, studies in other districts would likely result in similar findings due to not much differences in training, exposure to disability and cultural context among healthcare providers across the country, however future studies to compare possible differences between districts, should be undertaken. A further limitation is that research has clearly shown that individuals with certain types of disability, specifically those with intellectual impairments and those who are Deaf, may be at increased risk of having less contact with healthcare services because of communications barriers. In this study, we looked at disability overall, but the future study of access to healthcare among these sub-populations within the disability community is warranted. The ATDP survey instrument was designed to measure attitude towards disability in general (for all type of disabilities); therefore, the study reflects the healthcare provider’s views/attitude towards disability in general rather than a specific type. Additionally, we recruited women for the qualitative interview that had their last pregnancy within five years so we also recognize the potential for recall bias. Finally, we found that a number of women interviewed sought care from more than one health facilities at different level during their pregnancies; it is, therefore, unclear in some cases, which level of healthcare facilities the women interviewed were reporting on. In these cases, the response may reflect their cumulative experience and not be specific to a particular health facility or provider.

## Conclusion

Negative attitudes are prevalent towards women with disabilities, their pregnancy and maternal health needs among health providers in the study district. Interestingly, these attitudes are not universal, nor do they always translate into negative experiences by service users with disabilities although negative experiences are common. Inadequate public and professional knowledge about disability and needs of people with disabilities contributed to these negative attitudes.

Existing training courses and curricula designed for healthcare providers do not contain disability-related information or concerns. None of the healthcare providers in the study district was found to be trained on caring for or working with people with disabilities. However, a small number of providers attended disability orientation and sensitization sessions organized by NGOs. Provision of comprehensive training to maternal healthcare providers and sensitization training to all other health facility staff may help to improve maternal healthcare access for women with disabilities. In addition, disability-related questions should be included in the qualifying tests for healthcare providers including doctors, nurses and primary care providers at all levels.

This research revealed that healthcare providers in the study district had comparatively negative attitudes and behaviours towards people with disabilities. The results also indicated that women with disabilities using higher-level health facilities compared to those using community-level health facilities more commonly encountered negative attitudes and abusive behaviours. Existing literature provides some insights and evidence to help explain this, but access to health care services for women with disabilities remains an under-researched subject in Nepal and it deserves further exploration in order to assess and improve the effectiveness of current services and interventions designed to address attitudinal barriers.

As noted above, it can be concluded that lack of consistent and effective training for professionals means that their knowledge and attitudes towards disability often are no different from that of the general public. Specific training for healthcare professionals is urgently needed to ensure they are aware of how to appropriately address and work with people with disabilities in their professional capacities.

## Additional file

Additional file 1: Tables, File 2 Annex. (DOCX 31 kb)

## Abbreviations

AHW: Auxiliary Health Worker
AMDA: Association of Medical Doctors of Asia
ANC: Antenatal Care
ANM: Auxiliary Nurse Midwife
ANOVA: Analysis of variance
ATDP: Attitude towards disabled person
BLF: Big lottery fund
DPO: Disabled Person’s Organization
FCHV: Female community health volunteer
HA: Health assistant
HF: Health facility
HP: Health post
NGO: Non-government Organization
PHCC: Primary healthcare centre
SD: Standard deviation
SHP: Sub-health post
UHC: Urban health clinic
VDC: Village development committee
