# Supplementary material for: Healthcare provider’s attitude towards disability and experience of women with disabilities in the use of maternal healthcare service in rural Nepal
Source: Reprod Health. 2017 Jun 29;14:79. doi: 10.1186/s12978-017-0330-5 (PMC5492880; doi:10.1186/s12978-017-0330-5)
Supplement: Additional file 1: — Tables, File 2 Annex. (DOCX 31 kb) [file 12978_2017_330_MOESM1_ESM.docx]

## ANNEX 1: ATDP TOOL

**Attitude toward disabled persons (ATDP)**

**Survey tool adapted from the ATDP scale Form B**

By Yuker, Block & Young 1970

| **SN** | **Questions** | **-3** | **-2** | **-1** | **+1** | **+2** | **+3** |
| --- | --- | --- | --- | --- | --- | --- | --- |
| 1 | Disabled persons are usually friendly | 1. Disagree very much | 1. Disagree pretty Much | 1. Disagree a little | 1. Agree a little | 1. Agree pretty much | 1. Agree very much |
| 2 | People who are disabled should not have to pay income tax | 1. Disagree very much | 1. Disagree pretty Much | 1. Disagree a little | 1. Agree a little | 1. Agree pretty much | 1. Agree very much |
| 3 | Disabled people are no more emotional than other people | 1. Disagree very much | 1. Disagree pretty Much | 1. Disagree a little | 1. Agree a little | 1. Agree pretty much | 1. Agree very much |
| 4 | Disabled persons can have a normal social life | 1. Disagree very much | 1. Disagree pretty Much | 1. Disagree a little | 1. Agree a little | 1. Agree pretty much | 1. Agree very much |
| 5 | Most physically disabled persons have a chip on their shoulder | 1. Disagree very much | 1. Disagree pretty Much | 1. Disagree a little | 1. Agree a little | 1. Agree pretty much | 1. Agree very much |
| 6 | Disabled workers can be as successful as other workers | 1. Disagree very much | 1. Disagree pretty Much | 1. Disagree a little | 1. Agree a little | 1. Agree pretty much | 1. Agree very much |
| 7 | Very few disabled persons are ashamed of their disabilities. | 1. Disagree very much | 1. Disagree pretty Much | 1. Disagree a little | 1. Agree a little | 1. Agree pretty much | 1. Agree very much |
| 8 | Most people feel uncomfortable when they associate with disabled people | 1. Disagree very much | 1. Disagree pretty Much | 1. Disagree a little | 1. Agree a little | 1. Agree pretty much | 1. Agree very much |
| 9 | Disabled people show less enthusiasm than non-disabled people | 1. Disagree very much | 1. Disagree pretty Much | 1. Disagree a little | 1. Agree a little | 1. Agree pretty much | 1. Agree very much |
| 10 | Disabled people do not become upset any more easily than non-disabled people. | 1. Disagree very much | 1. Disagree pretty Much | 1. Disagree a little | 1. Agree a little | 1. Agree pretty much | 1. Agree very much |
| 11 | Disabled people are often less aggressive than normal people | 1. Disagree very much | 1. Disagree pretty Much | 1. Disagree a little | 1. Agree a little | 1. Agree pretty much | 1. Agree very much |
| 12 | Most disabled persons get married and have children | 1. Disagree very much | 1. Disagree pretty Much | 1. Disagree a little | 1. Agree a little | 1. Agree pretty much | 1. Agree very much |
| 13 | Most disabled persons do not worry any more than anyone else | 1. Disagree very much | 1. Disagree pretty Much | 1. Disagree a little | 1. Agree a little | 1. Agree pretty much | 1. Agree very much |
| 14 | Employers should not be allowed to fire disabled employees | 1. Disagree very much | 1. Disagree pretty Much | 1. Disagree a little | 1. Agree a little | 1. Agree pretty much | 1. Agree very much |
| 15 | Disabled people are not as happy as non-disabled ones | 1. Disagree very much | 1. Disagree pretty Much | 1. Disagree a little | 1. Agree a little | 1. Agree pretty much | 1. Agree very much |
| 16 | Severely disabled people are harder to get along with than are those with minor disabilities | 1. Disagree very much | 1. Disagree pretty Much | 1. Disagree a little | 1. Agree a little | 1. Agree pretty much | 1. Agree very much |
| 17 | Most disabled people expect special treatment | 1. Disagree very much | 1. Disagree pretty Much | 1. Disagree a little | 1. Agree a little | 1. Agree pretty much | 1. Agree very much |
| **SN** | **Questions** | **-3** | **-2** | **-1** | **+1** | **+2** | **+3** |
| 18 | Disabled persons should not expect to lead normal lives | 1. Disagree very much | 1. Disagree pretty Much | 1. Disagree a little | 1. Agree a little | 1. Agree pretty much | 1. Agree very much |
| 19 | Most disabled people tend to get discouraged easily | 1. Disagree very much | 1. Disagree pretty Much | 1. Disagree a little | 1. Agree a little | 1. Agree pretty much | 1. Agree very much |
| 20 | The worst thing that could happen to a person would be for him to be very severely injured | 1. Disagree very much | 1. Disagree pretty Much | 1. Disagree a little | 1. Agree a little | 1. Agree pretty much | 1. Agree very much |
| 21 | Disabled children should not have to compete with non-disabled children | 1. Disagree very much | 1. Disagree pretty Much | 1. Disagree a little | 1. Agree a little | 1. Agree pretty much | 1. Agree very much |
| 22 | Most disabled people do not feel sorry for themselves | 1. Disagree very much | 1. Disagree pretty Much | 1. Disagree a little | 1. Agree a little | 1. Agree pretty much | 1. Agree very much |
| 23 | Most disabled people prefer to work with other disabled people | 1. Disagree very much | 1. Disagree pretty Much | 1. Disagree a little | 1. Agree a little | 1. Agree pretty much | 1. Agree very much |
| 24 | Most severely disabled persons are not as ambitious as other people | 1. Disagree very much | 1. Disagree pretty Much | 1. Disagree a little | 1. Agree a little | 1. Agree pretty much | 1. Agree very much |
| 25 | Disabled persons are not as self-confident as physically normal persons | 1. Disagree very much | 1. Disagree pretty Much | 1. Disagree a little | 1. Agree a little | 1. Agree pretty much | 1. Agree very much |
| 26 | Most disabled persons don’t want more affection and praise than other people | 1. Disagree very much | 1. Disagree pretty Much | 1. Disagree a little | 1. Agree a little | 1. Agree pretty much | 1. Agree very much |
| 27 | It would be best if a disabled person would marry another disabled person | 1. Disagree very much | 1. Disagree pretty Much | 1. Disagree a little | 1. Agree a little | 1. Agree pretty much | 1. Agree very much |
| 28 | Most disabled people do not need special attention | 1. Disagree very much | 1. Disagree pretty Much | 1. Disagree a little | 1. Agree a little | 1. Agree pretty much | 1. Agree very much |
| 29 | Disabled persons want sympathy more than other people | 1. Disagree very much | 1. Disagree pretty Much | 1. Disagree a little | 1. Agree a little | 1. Agree pretty much | 1. Agree very much |
| 30 | Most physically disabled persons have different personalities than normal persons | 1. Disagree very much | 1. Disagree pretty Much | 1. Disagree a little | 1. Agree a little | 1. Agree pretty much | 1. Agree very much |

**Additional Questions**

| **SN** | **Question** | **Answers and code** | **Skip Pattern** |
| --- | --- | --- | --- |
| 1 | Have you provided services to disabled people?  (any type, any person – child or adult) | Yes……………………….…………1  No………………………….…..…..2 | GT 3 |
| 2 | Have you given maternal health care service to disabled pregnant women? | Yes………………………..………..1  No……………………..……………2 |  |
| 3 | Do you have any training on disability or treating/caring disabled person? | Yes…………………………….……1  No…………………………………..2 |  |

Thank You!
